# Camkii-Mediated Phosphorylation Regulates Distributions of Syngap-α1 and –α2 at the Postsynaptic Density

**DOI:** 10.1371/journal.pone.0071795

**Published:** 2013-08-13

**Authors:** Yijung Yang, Jung-Hwa Tao-Cheng, K. Ulrich Bayer, Thomas S. Reese, Ayse Dosemeci

**Affiliations:** 1 Laboratory of Neurobiology, National Institute of Neurological Disorders and Stroke, National Institutes of Health, Bethesda, Maryland, United States of America; 2 EM Facility, National Institute of Neurological Disorders and Stroke, National Institutes of Health, Bethesda, Maryland, United States of America; 3 Department of Pharmacology, University of Colorado Denver School of Medicine, Aurora, Colorado, United States of America; Centre national de la recherche scientifique, University of Bordeaux, France

## Abstract

SynGAP, a protein abundant at the postsynaptic density (PSD) of glutamatergic neurons, is known to modulate synaptic strength by regulating the incorporation of AMPA receptors at the synapse. Two isoforms of SynGAP, α1 and α2, which differ in their C-termini, have opposing effects on synaptic strength. In the present study, antibodies specific for SynGAP-α1 and SynGAP-α2 are used to compare the distribution patterns of the two isoforms at the postsynaptic density (PSD) under basal and excitatory conditions. Western immunoblotting shows enrichment of both isoforms in PSD fractions isolated from adult rat brain. Immunogold electron microscopy of rat hippocampal neuronal cultures shows similar distribution of both isoforms at the PSD, with a high density of immunolabel within the PSD core under basal conditions. Application of NMDA promotes movement of SynGAP-α1 as well as SynGAP-α2 out of the PSD core. In isolated PSDs both isoforms of SynGAP can be phosphorylated upon activation of the endogenous CaMKII. Application of tatCN21, a cell-penetrating inhibitor of CaMKII, to hippocampal neuronal cultures blocks NMDA-induced redistribution of SynGAP-α1 and SynGAP-α2. Thus CaMKII activation promotes the removal of two distinct C-terminal SynGAP variants from the PSD.

## Introduction

SynGAP is a ras GTPase activating protein (GAP) preferentially located in the postsynaptic density (PSD) of glutamatergic synapses [1]–[5]. SynGAP is involved in nervous system development and functions such as learning and memory, and mutations in this gene may result in nervous system pathology [6]–[10]. Previous studies in different laboratories have indicated an inhibitory function of SynGAP on the incorporation of AMPA receptors at the synapse, synaptic strength and spine growth [11], [12]. A recent study revealed that the effect of SynGAP on synaptic strength is isoform-specific: while overexpression of SynGAP-α1 isoforms have an inhibitory effect, overexpression of SynGAP-α2 isoforms enhances synaptic strength [13].

A major difference between SynGAP-α1 and SynGAP-α2 is that the former contains a C-terminal QTRV sequence that can bind to the PDZ domains of PSD-95 while the latter does not contain this sequence. In a previous immunogold electron microscopy study ([5], see corrigendum) we described activity-induced redistribution of SynGAP-α2 away from the PSD core. In the present study, we again use immunogold electron microscopy to compare the distribution patterns of SynGAP-α1 and SynGAP-α2 at the postsynaptic region of hippocampal neurons under basal conditions and following exposure to NMDA.

Activation of NMDA receptors promotes activation of Ca^2+^/calmodulin-dependent protein kinase II (CaMKII; reviews: [14], [15]). In turn CaMKII phosphorylates several PSD proteins including SynGAP [16], [17]. Here, we use tatCN21, a CaMKII-specific inhibitor peptide derived from the CaMKII inhibitor protein, CaMK-IIN [18]–[21], to examine and compare the possible role of CaMKII in the dynamics of SynGAP α1 and α2 isoforms.

## Materials and Methods

### Materials

Rabbit polyclonal antibody to the C-terminus of SynGAP-α1 (1∶2,500 for Western blots, 1∶50 for microscopy) was from Millipore (Billerica, MA) Rabbit monoclonal antibody (clone EPR2883Y) to the C-terminus of SynGAP-α2 (1∶2500 for Western blots, 1∶500 for microscopy) was from Millipore or Abcam (Cambridge MA). The two peptides KRLLDAQRGSFPPWVQQTRV and QITENGEFRNTADH (sequence verified with Epitomics, the originator of the monoclonal) used to generate SynGAP antibodies, corresponding to the C-termini of SynGAP-α1 (Q9QUH6-1 and Q9QUH6-3) and SyNGAP-α2 (Q9QUH6-4) respectively, do not have any common sequence motifs, thus making cross-reactivity improbable.

Rabbit polyclonal antibody to residues 290–307 [PRRYSPVAKDLLGEEDIC] of PSD-95 (1∶5000 for Western blots) was custom made by New England Peptide (Gardener, MA). N-methyl-D-asparic acid (NMDA) is from Tocris (Ellisville, MO). The CaMKII inhibitor tatCN21, a 21-amino acid peptide (CN21, amino acid sequence KRPPKLGQIGRSKRVVIEDDR) derived from CaMK-IIN [18] and fused to the cell-penetrating tat sequence, is more effective than KN-93 in inhibiting both Ca^2+^-dependent and Ca^2+^-independent activity of CaMKII and is specific for CaMKII [19]–[21]. The control peptide (tatCtrl) used in this study is the tat sequence fused to a scrambled sequence of CN21 (VKEPRIDGKPVRLRGQKSDRI) [21].

### Preparation and Treatment of PSD Fractions

PSD fractions were prepared as described previously [22] from adult rat brains collected and frozen in liquid nitrogen within 2 minutes of decapitation by Pel-Freeze Biologicals (Rogers, AR). PSD fractions were pre-incubated in 0.1 M DTT on ice for two hours before incubation in phosphorylation buffer. Endogenous phosphorylation of PSD proteins was performed by incubation of PSD fractions (0.4 mg/mL final protein concentration) for 15 minutes at 37°C in phosphorylation buffer which contained 1 mM CaCl_2_ and 40 µg/mL calmodulin (or 1 mM EGTA), 5 mM MgCl_2_, 100 µM ATP, 50 µg/mL leupeptin, 20 mM DTT, 0.4 µM Microcystin-LR, 20 mM HEPES, pH 7.4). CaMKII inhibitor and control peptides were included at the final concentration of 20 µM. The reaction was stopped by addition of SDS-containing PAGE sample buffer for Western analysis.

### Western Immunoblot

Samples were separated by SDS-PAGE on 4–15% gradient gels from BioRAD and transferred to PVDF membranes, which were incubated with specified primary antibodies and then with horseradish peroxidase-conjugated secondary antibodies (1∶50,000 dilution) and visualized by chemiluminescence (SuperSignal West Pico, Thermo Scientific). The two bands (around 150 kDa for SynGAP and just below 100 kDa for PSD-95) are a sufficient distance apart to allow for the membrane to be cut into two pieces and immunoblotted for SynGAP and PSD-95 independently, allowing simultaneous visualization of these two bands on the same lane. Immunoblots in figures illustrate upper and lower portions, which were probed with anti-SynGAP and anti-PSD-95, respectively.

### Preparation and Treatment of Dissociated Hippocampal Cultures

#### Ethics statement

The animal protocol was approved by NIH Animal Use and Care Committee and conformed to NIH guidelines. Hippocampal cells from 21-day embryonic Sprague-Dawley rats were dissociated and grown on a glial cell layer as described previously [23] for 19–21 days. Cell cultures were treated as described previously [5]. Culture dishes were removed from the incubator and washed once with normal (control) incubation medium (124 mM NaCl, 2 mM KCl, 1.24 mM KH_2_PO_4_, 1.3 mM MgCl_2_, 2.5 mM CaCl_2_, 30 mM glucose in 25 mM HEPES at pH 7.4) maintained at 37°C. Experimental treatment of cell cultures was performed on a floating platform in a water bath maintained at 37°C. Peptides, 20 µM tatCN21 or 20 µM tatCtrl, and 50 µM NMDA were added to the normal medium as indicated. Pre-incubation for 20 min in the presence or absence of tat-peptides was followed by 2 minutes incubation in the same media with or without NMDA.

### Pre-embedding Immunogold-labeling

After treatment, neuronal cultures were processed for pre-embedding immunogold-labeling as described previously [5]. Briefly, cultures were fixed in 4% paraformaldehyde (EMS, Hatfield, PA) in PBS for 25–45 min at room temperature. Samples were permeabilized (0.1% saponin for 40–60 min) and incubated with primary and secondary antibodies for 1–1.5 hr (Nanogold, Nanoprobes, Yaphank, NY). Samples were then fixed with 2% glutaraldehyde in PBS, silver enhanced (HQ kit, Nanoprobes), and processed for electron microscopy. Only parallel samples from the same experiment were directly compared because the overall labeling sensitivity may differ between experiments.

### Morphometry and Statistical Analysis

Synaptic profiles were photographed with a JEOL electron microscope with a CCD camera (XR-100 from AMT, Danvers, MA, USA). The PSD complex was defined as the PSD core and contiguous network (40–120 nm from the postsynaptic membrane), as explained previously [5]. Distance of SynGAP label from the postsynaptic membrane was measured from the center of each label to the cleft edge of the postsynaptic membrane using ImageJ (National Institutes of Health, Bethesda, MD, USA). To quantify movement of SynGAP out of the core of the PSD upon stimulation, the distance measurements were plotted as histograms. Because the distribution of SynGAP at the PSD is skewed, a non-parametric comparison of the median values -Wilcoxon-Mann-Whitney rank sum test (KaleidaGraph, Synergy Software, Reading, PA)- was applied. Statistical significance was determined with Bonferroni adjusted alpha level of 0.005 per test (0.05/10).

## Results

### SynGAP-α1 and SynGAP-α2 are Enriched in PSD Fractions

**Figure 1:**
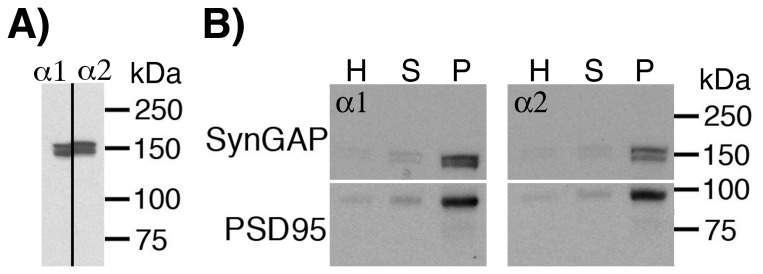
SynGAP-α1 and SynGAP-α2 in the PSD fraction. A: Antibodies for SynGAP-α1 and SynGAP-α2 recognize doublets with distinct mobilities. Immunoblots of a PSD fraction where a single lane was cut into half (vertical line), and left and right halves were probed with anti-SynGAP-α1 and anti-SynGAP-α2 respectively. B: PSD fractions are highly enriched in SynGAP-α1 and SynGAP-α2. Western blots of the subcellular fraction from brain probed with anti-PSD-95 (bottom portion of membranes) or anti-SynGAP-α1 or anti-SynGAP-α2 (upper portion of membranes). The white horizontal line denotes where the membrane was cut for immunoblotting with different antibodies. H: Homogenate; S: synaptosome; P: PSD.

Antibodies raised to peptides with sequences corresponding to the C-termini of SynGAP-α1 and SynGAP-α2, each recognized a doublet with close but distinct electrophoretic mobilities (Fig. 1 A), as reported previously [13]. Comparison of the PSD fraction with the parent fractions revealed enrichment of both SynGAP-α1 and SynGAP-α2 in the PSD fractions that was comparable to the enrichment of the PSD marker protein PSD-95 (Fig. 1 B).

### CaMKII Mediates the Ca^2+^/calmodulin-dependent Phosphorylation of SynGAP-α1 and SynGAP-α2 at the PSD

**Figure 2:**
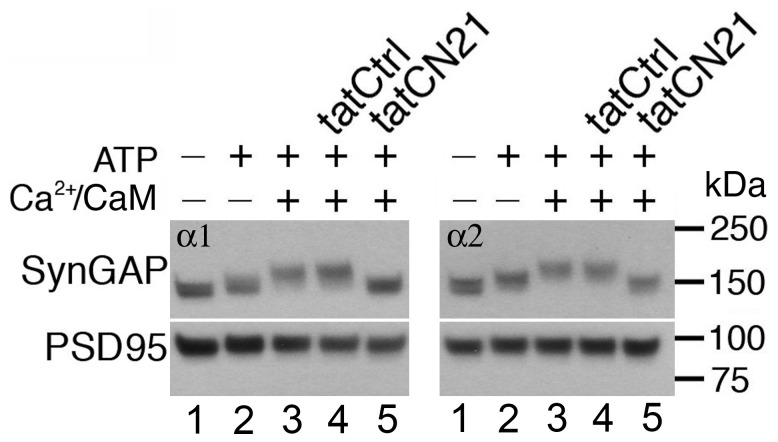
CaMKII mediates phosphorylation of SynGAP-α1 and SynGAP-α2. Isolated PSD fractions were incubated under the conditions indicated. Phosphorylation was assessed by characteristic changes in mobility. Both SynGAP-α1 and SynGAP-α2 show a small shift in mobility upon incubation with ATP in the absence of Ca^2+^ (1 mM EGTA). A further shift is observed upon addition of Ca^2+^/calmodulin (CaM). This shift is reversed by the addition of the CaMKII inhibitor tatCN21, while the control peptide, tatCtrl, has no effect. The white horizontal lines denote the position of the cut in the membrane for immunoblotting with different antibodies.

Isolated PSDs were incubated under conditions designed to manipulate the activity of endogenous CaMKII. Inclusion of ATP during incubation of isolated PSDs in Ca^2+^/calmodulin-free phosphorylation buffer led to a shift in electrophoretic mobilities of SynGAP-α1 and SynGAP-α2 (Fig. 2, lanes 1 vs 2), implying phosphorylation by a kinase activity that does not require Ca^2+^/calmodulin. Incubation of PSDs in medium containing Ca^2+^, calmodulin, and ATP led to a further shift in the mobilities of SynGAP-α1 and SynGAP-α2 (Fig. 2, lanes 2 vs 3). These distinct stepwise shifts in the mobilities of SynGAP-α1 and SynGAP-α2 point to phosphorylation of distinct sites by Ca^2+^-independent and Ca^2+^-dependent kinase activities.

A specific inhibitor of CaMKII, tatCN21, was used to test the involvement of CaMKII in the phosphorylation of SynGAP isoforms. Inclusion of tatCN21 (20 µM) during incubation in the phosphorylating medium containing Ca^2+^/calmodulin returned the electrophoretic mobilities of SynGAP-α1 and SynGAP-α2 to those observed in the absence of Ca^2+^/calmodulin (Fig. 2, lanes 3 vs 5). In contrast, addition of a control peptide (tatCtrl) had no effect on the mobilities of either SynGAP isoform (Fig. 2, lanes 3 vs 4). These results indicate that CaMKII mediates the Ca^2+^/calmodulin-dependent phosphorylation of both SynGAP-α1 and SynGAP-α2 at the PSD. Indeed, SynGAP-α1 and SynGAP-α2 both contain the three sequences previously described to be phosphorylated upon activation of CaMKII in isolated PSDs [17]. Phosphorylation in the absence of Ca^2+^, on the other hand, is not reversed by tatCN21, indicating that it is mediated by a kinase other than the autonomous form of CaMKII, since tatCN21 inhibits Ca^2+^-dependent as well as Ca^2+^-independent forms of CaMKII [20].

### CaMKII Activity is Required for NMDA-induced Redistribution of SynGAP-α1 and SynGAP-α2

**Figure 3:**
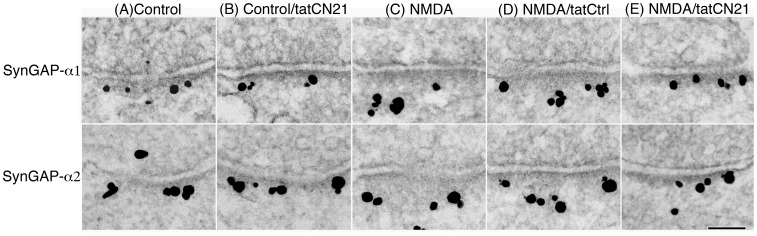
Inhibition of CaMKII blocks NMDA-induced redistribution of SynGAP-α1 and SynGAP-α2. Electron micrographs of hippocampal neuronal cultures immunogold labeled for SynGAP-α1 or SynGAP-α2 show that label (black grains) for both isoforms is close to the postsynaptic membrane under control conditions (A&B). Following 2 min exposure to 50 µM NMDA, label moves away from the postsynaptic membrane (C). Inclusion of tatCN21, a CaMKII inhibitor, inhibits NMDA-induced redistribution of SynGAP away from the PSD core (E) whereas inclusion of a control peptide, tatCtrl, has no effect (D). Scale bar = 100 nm.

Using tatCN21, the role of CaMKII in the distribution of SynGAP-α1 and SynGAP-α2 at the PSD complex was examined by pre-embedding immunogold electron microscopy in dissociated hippocampal cultures in the presence or absence of tatCN21. Hippocampal cultures were preincubated for 20 min in the presence or absence of tatCN21 before exposure to NMDA. Fig. 3 shows representative electron micrographs of the synaptic region following different treatment conditions. Under basal conditions, label for both SynGAP-α1 and SynGAP-α2 was localized near the postsynaptic membrane (Fig. 3 A&B). Application of NMDA led to redistribution of SynGAP-α1 and SynGAP-α2 label further away from the postsynaptic membrane (Fig. 3 C). This NMDA-induced redistribution of SynGAP was blocked by tatCN21 (Fig. 3 E) whereas the control peptide tatCtrl showed no appreciable effect (Fig. 3 D).

### Measuring of the Effect of tatCN21 on NMDA-induced Redistribution of SynGAP-α1 and SynGAP-α2

**Figure 4:**
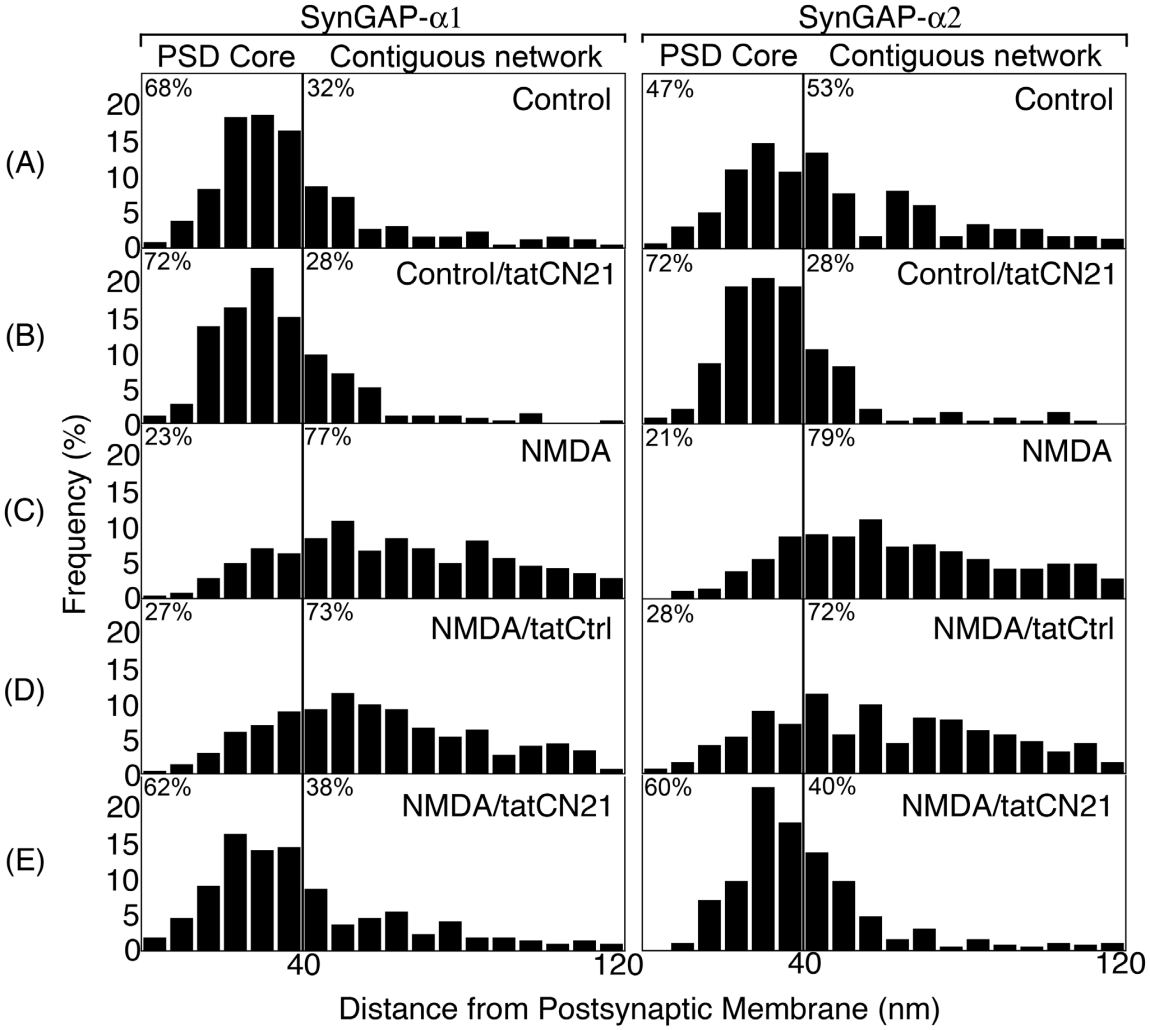
Distribution of SynGAP-α1 and SynGAP-α2 within the PSD complex is regulated by CaMKII activity. Histograms of distance measurements from representative experiments (Table 1: Exp1 & Exp 4) showing the distribution of label for two SynGAP isoforms under different treatment conditions. The vertical line denotes the boundary between the PSD core and the contiguous network. The percents of label in the PSD core and contiguous network are indicated on either side of the boundary line in each box. Under control conditions, distribution of label for both SynGAP-α1 and SynGAP-α2 are predominantly in the PSD core (A&B). Upon application of NMDA, the label for both isoforms shifts away from the PSD core (C). Presence of tatCN21 during treatment with NMDA inhibits redistribution (E), whereas the control peptide tatCtrl has no effect (D).

Distances of SynGAP labels from the postsynaptic membrane were measured in samples subjected to different experimental conditions as described in Fig. 3 and displayed as histograms (Fig. 4). The PSD complex was divided into two zones, the PSD core and the contiguous network (Methods). In control samples, significant SynGAP-α1 and SynGAP-α2 label (Fig. 4 A) was located within the PSD core, an area extending up to 40 nm from the postsynaptic membrane. Inhibition of CaMKII caused a further shift in SynGAP label toward the PSD core. After application of tatCN21 for 20 min at least 70% of either SynGAP-α1 or SynGAP-α2 label was within the PSD core (Fig. 4 B). Treatment with NMDA promoted a shift in the distribution of SynGAP-α1 and SynGAP-α2 label out of the PSD core, with only a minor fraction remaining (Fig. 4 C). The NMDA-induced shift in the redistribution of SynGAP label was prevented by tatCN21 (Fig. 4 E). It should be noted that the figure illustrates the distribution of SynGAP label within the PSD complex as a percentage of the total label within that compartment.

Median values for distances of labels from the postsynaptic membrane under the five experimental conditions are shown in Table 1. Median values rather than mean values were used for comparison due to the skewed distribution of SynGAP under basal conditions. Results from two independent experiments with each antibody are tabulated. Differences between control and NMDA-treated samples (Table 1: 1^st^ vs 3^rd^ columns) were statistically significant in all four experiments, indicating that NMDA induces redistribution of both SynGAP-α1 and SynGAP-α2. Inclusion of tatCN21 before and throughout NMDA treatment inhibited the NMDA effect. The differences between samples treated with NMDA and NMDA plus tatCN21 (Table 1: 3^rd^ vs 5^th^ columns) were statistically significant in all four experiments. The control peptide (tatCtrl) had no statistically significant effect on the median distance in any experiment, although a small reduction was consistently observed (Table 1: 3^rd^ vs 4^th^ columns). These results demonstrate an involvement of CaMKII in mediating NMDA-induced movements of SynGAP-α1 and SynGAP-α2 away from the postsynaptic membrane. Upon application of tatCN21 in the absence of NMDA, the median distances of SynGAP-α1 as well as SynGAP-α2 labels from the postsynaptic membrane showed a decrease (Table 1: 1st vs 2nd columns). The decreases assessed in individual experiments were statistically significant except in experiment 1 for SynGAP-α1. This observation suggests that even in the absence of pharmacological stimulation, the activity level of CaMKII in neurons impacts the distribution of SynGAP at the PSD.

**Table 1:** Median distance of SynGAP label from the postsynaptic membrane (nm) under different treatment conditions.

Experiment (Antibody)	1:Control (n)	2:Control/tatCN21 (n)	3:NMDA (n)	4:NMDA/tatCtrl (n)	5:NMDA/tatCN21 (n)
Exp1 (SynGAP-α1)	33.1 (260)	30.7 (294)	60.5 (277)	55.1 (303)	35.3 (214)
Exp2 (SynGAP-α1)	38.7 (41)	25.2 (97)	84.6 (37)	64.0 (97)	34.7 (42)
Exp3 (SynGAP-α2)	41.7 (236)	33.3 (315)	60.1 (266)	53.1 (354)	36.5 (409)
Exp4 (SynGAP-α2)	41.7 (290)	32.5 (242)	59.8 (282)	56.7 (324)	36.5 (390)

With each antibody, two independent experiments were carried out.

In each experiment, the median distances of gold label from the postsynaptic membrane were compared among five groups (sister cultures subjected to different incubation conditions). Sample size (n) represents the total number of gold labels pooled from all synapses sampled under each condition.

## Discussion

Comparison of subcellular fractions from brain showed marked enrichment of SynGAP-α1 and SynGAP-α2 in PSD fractions, implying that both isoforms are part of the PSD complex. Immunoelectron microscopy using isoform-specific antibodies allowed precise localization of both isoforms in the PSD complex, revealing that SynGAP-α1 (which contains a PDZ-binding C-terminal QTRV sequence) and SynGAP-α2 (which does not) have similar distributions. Under basal conditions, especially when CaMKII activity is blocked, both SynGAP-α1 and SynGAP-α2 are mostly localized within the PSD core. These results suggest that the unique QTRV domain of SynGAP-α1 is not necessary for its localization at the PSD core. We speculate that the association of the QTRV sequence of SynGAP-α1 with the PDZ domain of PSD-95 serves a distinct function, that of blocking the association of other proteins with PSD-95. Indeed, several other proteins bind to the same region of PSD-95, including TARPs, the auxiliary proteins of AMPA receptors (review: [24]).

Similar to the distribution pattern of SynGAP-α2 described previously [5], the distribution pattern of SynGAP-α1 at the PSD complex changes drastically upon excitation. Following application of NMDA for two minutes, significant fractions of both isoforms move out of the PSD core. NMDA-induced movement of both SynGAP isoforms reverses within 30–45 min of return to normal medium (Tao-Cheng unpublished observations). Acute global NMDA treatment of neuronal cultures has been previously shown to increase the levels of autonomous (phosphoThr286) CaMKII, to cause CaMKII translocation to the PSD [25] and to promote CaMKII-mediated phosphorylation of SynGAP [16]. Thus, in the present study, we explored the involvement of CaMKII-mediated phosphorylation in the movement of SynGAP isoforms induced by NMDA. Using tat CN21, a specific inhibitor of CaMKII [19], we demonstrate that both SynGAP-α1 and SynGAP-α2 can be phosphorylated upon activation of the endogenous CaMKII in isolated PSDs. Furthermore, preincubation of neurons with tatCN21 blocks NMDA-induced redistribution of both SynGAP isoforms. In fact, CaMKII activity must control the distribution of SynGAP even under basal conditions, because application of tatCN21(which blocks autonomous as well as Ca^2+^-dependent CaMKII activity) in normal medium results in more SynGAP label in the PSD core. In addition to some autonomous CaMKII that may be present in resting neurons, spontaneous neuronal firing is likely to contribute to the basal CaMKII activity levels. Thus, the activity of CaMKII is a major determinant of SynGAP distribution at the PSD. We attribute the difference between the median distances of SynGAP-α2 from the postsynaptic membrane in the present study (42 nm) and our previous study (34 nm) [5] to different basal CaMKII activities in different batches of cultured hippocampal neurons.

NMDA-induced, CaMKII-mediated movement of SynGAP out of the PSD core can have multiple functional consequences. One range of possibilities is related to the displacement of GAP activity. Ras-regulating GAP activity is required for the inhibitory effect of SynGAP-α1 on synaptic transmission [12]. Activity-induced redistribution of SynGAP would change the access of the protein to Ras and thus may constitute a step in activity-induced synaptic modification. Indeed, Araki and Huganir, using live imaging techniques, report NMDA receptor- and CaMKII-mediated exclusion of SynGAP-α1 from spines and concomitant activation of Ras following a chemical LTP protocol ([26] and personal communication with R. Huganir). In the present study we observed movement of SynGAP by EM immunolabeling in a confined region that covers the PSD complex. Our results show a distinct movement of both SynGAP-α1 and SynGAP-α2 out of the PSD core upon application of NMDA. Movement of SynGAP within the spine versus exclusion of the protein from the spine are likely to have opposite functional consequences, as the former would tend to make SynGAP more accessible to soluble Ras in the spine cytoplasm, whereas the latter would prevent its access.

Another likely consequence of SynGAP-α1 movement out of the PSD core is dissociation of the C-terminal QTRV sequence from the PDZ domain of PSD-95. Previous studies support the idea that the QTRV sequence of SynGAP-α1 is also required for the inhibitory effect of the protein on synaptic transmission. While overexpression of SynGAP-α1 reduces mEPSCs from AMPA receptors, overexpression of a mutant lacking the QTRV sequence fails to do so [12]. As discussed above, it is possible that the inhibitory effect of SynGAP-α1 on AMPA receptor-mediated functions is at least partly due to its ability to block anchoring of the receptors through association with PSD-95. In this case, movement of a SynGAP-α1 molecule out of the PSD core would empty a slot for the association of an AMPA receptor. A recent study demonstrated that overexpression of SynGAP with α1-type C-terminus containing a QTRV sequence reduces mEPSCs mediated by AMPA receptors, while the overexpression of SynGAP with α2-type C-terminus without the QTRV sequence enhances these mEPSCs [13]. Our observation that the two isoforms occupy the same locations within the PSD core raises the possibility that overexpression of one isoform would displace the other and thus promote changes in synaptic strength.

NMDA receptor activation has been implicated in the induction of LTP as well as LTD in the same synapses in the hippocampus ([27], [28], review: [29]). The NMDA-induced, CaMKII-mediated movement of both SynGAP isoforms away from the PSD core shown here may constitute a first common step towards either an increase or a decrease in synaptic strength. If the ratio of SynGAP-α1 to SynGAP-α2 at the PSD determines the number of available slots for AMPA receptors and synaptic strength, CaMKII-mediated removal of both SynGAP isoforms from the PSD core would produce a window of opportunity for modification of synaptic strength in either direction.
